# The prevalence trend of metabolic syndrome and its components and risk factors in Korean adults: results from the Korean National Health and Nutrition Examination Survey 2008–2013

**DOI:** 10.1186/s12889-016-3936-6

**Published:** 2017-01-13

**Authors:** Binh Thang Tran, Bo Yoon Jeong, Jin-Kyoung Oh

**Affiliations:** 1 Department of Cancer Control and Policy, Graduate School of Cancer Science and Policy, 323 Ilsan-ro, Ilsandong-gu, Goyang-si, Gyeonggi-do, 410-769 Republic of Korea; 2 Cancer Risk Appraisal & Prevention Branch, National Cancer Center, 323 Ilsan-ro, Ilsandong-gu, Goyang-si, Gyeonggi-do, 410-769 Republic of Korea

**Keywords:** Metabolic syndrome, Prevalence, KNHANES

## Abstract

**Background:**

Abnormalities in the clinical markers of metabolic syndrome (MS) are associated with the development of cardiovascular disease, type 2 diabetes mellitus, and some cancers. MS prevalence in Korea increased between the mid-1990s and mid-2000s; however, no data on the recent trends of MS prevalence are available. Thus, we aimed to investigate the prevalence of MS, the five components of MS, and the related risk factors in Korean adults by using recent data.

**Methods:**

Data from the Korean National Health and Nutrition Examination Survey conducted between 2008 and 2013 were used. The revised National Cholesterol Education Program criteria were used for defining MS. A multivariate logistic regression analyses was used to estimate the relationship between the related risk factors including behaviors, dietary factors, and the prevalence of MS.

**Results:**

A total of 34,587 men and women were included in the analysis. Age-adjusted prevalence of MS in 2013 was 28.9% without a significant increasing or decreasing trend between 2008 and 2013. Among the five components of MS, abdominal obesity decreased in both men and women (annual percent change: −2.0 and −2.5%, respectively), the decrease being significant only in women, whereas blood pressure and blood glucose significantly increased in men (+1.9 and +2.7%, respectively). Age and obesity (odds ratio = 6.7, 95% confidence interval = 5.9–7.5 for body mass index ≥25 kg/m^2^ vs. body mass index <25 kg/m^2^) were associated with increased MS risk in both men and women. Smoking and alcohol drinking were significantly associated with increased MS risk in men, and association between MS and vitamin D deficiency was at the edge of statistical significance. Higher education and income level were significantly associated with decreased MS risk in women. During this period, smoking rate and physical activity, sodium intake, and serum vitamin D level significantly decreased. Education level, calorie intake, and intake of carbohydrate, fat, protein and calcium significantly increased.

**Conclusion:**

Several factors contribute to the stable MS prevalence—on the one hand, increased prevalence of high blood sugar, high blood pressure, calorie intake, and physical inactivity, and on the other hand, decreased prevalence of abdominal obesity and smoking. Lifestyle interventions to prevent and control non-communicable diseases should be implemented at the national level to reduce the burden of MS.

## Background

Metabolic syndrome (MS) is a common metabolic disorder defined as a cluster of metabolic abnormalities characterized by the co-occurrence of at least three of the following criteria,: hypertension, high triglyceride (TG) levels, low high-density lipoprotein-cholesterol (HDL-C) levels, abdominal obesity, and high fasting plasma glucose (FPG) [1]. These factors contribute to the increased risk of type 2 diabetes mellitus, cardiovascular diseases (CVD), and all-cause mortality. MS also is associated with a high risk of colorectal, pancreatic, and breast cancers [2]. Other criteria measurements of MS could also be used such as the following: proinflammatory state (elevated C-reactive protein [CRP]), prothrombotic state (increased plasma plasminogen activator inhibitor (PAI)-1 and fibrinogen), elevated small low-density lipoprotein (LDL) level, physical inactivity, high resting heart rate, and low heart rate variability. The prevalence of MS was approximately 35% in the adult population of United States during 2003–2012, and 50% of those aged 60 years or older were estimated to have MS [3]. MS prevalence was found to be 24.3, 8.4, and 24.5% in 10 European countries [4], Japan [5], and China [6], respectively.

In Korea, major causes of death are non-communicable diseases, comprising cancers, CVD, diabetes, and chronic lung diseases [7]. These diseases are preventable by modifying behavioral or intermediate risk factors like hypertension, pre-diabetic status, obesity, and MS.

According to a different definition of MS, its prevalence in Korea 2001 was 1.6–29.6% in men and 10.1–32.8% in women [8]. The economic burden of MS-related cancers in 2012 was USD 3.32 billion and that attributable to MS was USD 199.8 million, accounting for 6.0% of the total cost including direct and indirect cost in Korea [9]. These findings indicate that the increasing prevalence of MS should be addressed in terms of cancer prevention and public health intervention programs. The prevalence of MS, like those of chronic diseases, has been rising steadily worldwide, and Korea is no exception. Well-known factors such as aging, increased life expectancy, increased body fatness, and westernized diet contribute to the prevalence of MS in Korea. In order to develop strategies for MS prevention, the trend in the prevalence of MS and its components and risk factors needs to be characterized among a representative Korean population.

We, therefore, report on the prevalence and trend of MS and five of its components, as well as the related risk factors including behaviors and dietary factors in Korean adults aged at least 20 years by using the Korean National Health and Nutrition Examination Survey (KNHANES) data from 2008 to 2013.

## Methods

### Design and data collection

This study was based on 6-year data obtained from the KNHANES survey carried out from 2008 to 2013. The details about the survey have been published elsewhere [10]. In brief, KNHANES is an annual surveillance system that uses a representative national sample and consists of three surveys, health interview survey, health examination survey, and nutrition survey, which collectively assess the health and nutritional status of the Korean population. The survey has been conducted by the Korea Centers for Disease Control and Prevention.

A stratified multistage probability sampling method was used, with selections made from sampling units based on geographical area, sex, and age groups using household registries. The households were screened for eligible persons; those aged ≥1 year were selected from each household agreeing to participate. Each stage was generated to reflect the probabilities of selection by using sampling weights. Among 40,328 participants aged ≥20 years, 34,587 individuals who participated in the survey during 2008–2013 and had all the data required for defining MS according to the revised National Cholesterol Education Program (NCEP) definition were included in the present study. Written informed consent was obtained from all participants.

### Health examination and interview

In the health interview, participants were asked to provide information about age, education, smoking history, and alcohol intake. Height and weight measurements were performed with the participants wearing light clothing and no shoes. Those with a body mass index (BMI) ≥25 kg/m^2^ were classified as obese [11]. Waist circumference was measured from the narrowest point between the lower border of the rib cage and the iliac crest.

To overcome a systematic error in blood pressure (BP) measurements caused by the height of the forearm in the survey conducted between 2008 and 2010, we calculated adjusted BP according to the mean height of the arm at the level of the heart and the mean of the second and third measurements.

Smoking status was divided into two categories: no smoking and current smoking. Alcohol consumption was classified as current drinking and no drinking. Physical activity was measured by using the short version of the International Physical Activity Questionnaire and was divided into the following four categories: inactive, walking, moderate physical activity, and high physical activity [12]. Monthly household income was categorized into quartiles (lowest, medium-low, medium-high, and highest) according to the equivalent household income (equivalent household income = monthly household income/square root of the number of household members, after considering sex and each five-year age stratum). Education level was divided into elementary school, middle school, high school, and college or higher.

### Assessment of nutrient intake

The usual dietary habits were ascertained by the 24-h recall method before assessing dietary intake, and nutrient intake was estimated using the Can-Pro 2.0 nutrient intake assessment software developed by the Korean Nutrition Society. Dietary intake information was obtained via a face-to-face interview conducted using a validated semi-quantitative food frequency questionnaire for Koreans, and it took into consideration the participant’s consumption of 63 food items [13]. We excluded those with a calorie intake ≤500 Kcal or ≥4000 Kcal because of implausible energy intakes. The nutrient variables were used as continuous data of daily intake of total calories (kcal), carbohydrates (g/dL), fiber (g/dL), sodium (mg/dL), protein (mg/dL), fiber (mg/dL), vitamin C (mg/dL), fat (g/dL), and calcium (mg/dL). These variables were found to be significantly associated with MS [14–18].

### Blood sampling and blood assay

Blood samples were collected from the antecubital vein after more than eight h of fasting and analyzed at a central certified laboratory to determine serum concentration of fasting glucose. HDL-C, TG, fasting plasma glucose (FPG), and insulin levels were measured using enzyme or radioimmunoassay methods. All samples were processed according to the KNHANES protocol [19].

Because the analysis tool and the method of measuring HDL-C were altered due to changes by the clinical laboratory organization, revised HDL-C level was derived since 2007 according to the Lipid Standardization Program released by the Centers for Disease Control and Prevention in the United States [20]. The revised HDL-C extrapolations were based on the regression line, and it may theoretically cause an excess residual error for prediction.

The revised HDL cholesterol was calculated as follows [21]:

$$ \mathrm{Revised}\ \mathrm{H}\mathrm{D}\mathrm{L}\ \mathrm{cholesterol}\ \left(2008\hbox{--} 2011\right):\ {\mathrm{HDL}}_{\mathrm{adjusted}} = 0.872 \times \mathrm{H}\mathrm{D}\mathrm{L} + 2.460 $$

$$ \mathrm{Revised}\ \mathrm{H}\mathrm{D}\mathrm{L}\ \mathrm{cholesterol}\ \left(2012\hbox{--} 2013\right):\ {\mathrm{HDL}}_{\mathrm{adjusted}} = 0.952 \times \mathrm{H}\mathrm{D}\mathrm{L} + 1.096 $$

Vitamin D deficiency was defined as a 25-hydroxyvitamin D serum concentration ≤20 ng/mL.

### Definition of MS

The definition provided by the modified Third National Cholesterol Education Program Expert Panel on Detection, Evaluation, and Treatment of High Blood Cholesterol in Adults (NCEP-ATP III) and the specific values for waist circumference provided by the World Health Organization and the Korean Society for the Study of Obesity were used to determine MS and its components [1] [22]. MS was diagnosed if three or more of the following criteria were met: (i) abdominal obesity (men >90 cm and women >85 cm); (ii) elevated BP: systolic BP ≥130 mm Hg and/or diastolic BP ≥85 mm Hg or currently undergoing treatment for hypertension; (iii) elevated TGs: TGs ≥150 mg/dL or current drug treatment for high TGs; (iv) reduced HDL-C (men <40 mg/dL and women <50 mg/dL) or current drug treatment for low HDL-C; and (v) elevated fasting glucose: FPG ≥100 mg/dL or current treatment with a hypoglycemic agent or insulin. Those who fasted <8 h or were pregnant were excluded while estimating the prevalence of MS.

### Statistical analyses

The chi-square test and ANOVA were used to compare differences between participant characteristics, risk factors, MS components, and time. Age-standardized prevalence was estimated by the direct method using the 2005 Korean census population as the standard. The sampling weight method was used to assign participants as representatives of the Korean population. We calculated *P for trend* using the quantile regression model or logistic regression model, both of which were age-adjusted.

The multivariate logistic regression model was used to estimate the predictive factors contributing to an increased prevalence of MS. We used the Bayesian model average (BMA) for selecting appropriate models. BMA provides an approach that takes into account model uncertainty by combining information from a pre-determined subset of all possible models and obtaining a weighted average of the quantity of interest over these models and lists the five best models [23]. In the BMA model, we included all risk factors stratified by sex such as age, BMI (≥25 kg/m^2^), income (quartiles), current smoking (yes/no), drinking (yes/no), physical activity (yes/no), intake of total calories (kcal), carbohydrates (g/dL), fiber (g/dL), calcium (mg/dL), sodium (mg/dL), protein (g/dL), fiber (g/dL), vitamin C (mg/dL), and fat (g/dL), and vitamin D deficiency. Variables that were less important had smaller weights. Therefore, we chose the best model with five variables among men (age group, obesity (BMI, ≥25 kg/m^2^), drinking, smoking, and vitamin D deficiency) with the highest posterior model probability in the final analysis. Among women, the best posterior model probability included variables such as age group, obesity (BMI, ≥25 kg/m^2^), income (quartiles), and education.

The odds ratio (OR) and 95% confidence interval (CI) were used to estimate the association between all risk factors selected from the BMA model as independent variables, and MS was examined as a dependent variable. All analyses were performed using STATA (version 13.0) and R-language statistical software.

## Results

Table 1 shows the descriptive statistics of the characteristics, health behavior, nutrient factors, and anthropometric and biochemical parameters of participants during the survey period 2008–2013. Smoking rate decreased from 25.7% in 2008 to 23.2% in 2013 (*P* for trend < 0.01). Physical activity also decreased from 56.7% in 2008 to 45.5% in 2013 (*P* for trend < 0.001), especially in the walking and moderate level of activity. Prevalence of obesity (BMI ≥25.0 kg/m^2^) remained stable. Calorie intake and nutrient intake including carbohydrate, fat, protein, and calcium significantly increased, whereas sodium intake and serum vitamin D level significantly decreased.

**Table 1:** Age-adjusted anthropometric, nutrient intake, and biochemical parameters from 2008 to 2013

Year	2008	2009	2010	2011	2012	2013	*p-*value	*P* for trend
*N* = 34,587	6,215	6,872	5,661	5,667	5,263	4,909		
Age (years)	49.1 ± 0.2	49.1 ± 0.2	49.3 ± 0.2	50.9 ± 0.2	51.4 ± 0.2	49.2 ± 0.2	<0.001	
Male (%)	41.7	43.5	43.2	42.6	41.8	43.1	0.201	
Income (Quartile)							<0.001	0.38
* Q1*_*lowest*_	16.0	16.9	17.5	15.5	13.8	15.4		
* Q2*_*medium-lowest*_	26.6	22.6	26.7	28.2	26.5	25.8		
* Q3*_*Medium highest*_	27.9	29.5	29.8	29.7	29.1	28.3		
* Q4*_*highest*_	29.5	31.0	26.0	26.6	30.6	30.5		
Education							<0.001	<0.001
Elementary school	19.6	18.9	18.6	18.0	17.6	15.9		
Middle school	11.1	10.4	10.2	10.7	9.0	9.1		
High school	39.2	40.4	36.8	37.3	40.6	38.6		
College and over	30.1	30.3	34.4	34.0	32.8	36.4		
Smoking (%)^a^	25.7 ± 0.7	25.8 ± 0.7	25.2 ± 0.9	24.9 ± 0.9	23.6 ± 0.9	23.2 ± 0.9	0.006	<0.01
Alcohol drinking (%)^a^	11.5 ± 0.5	12.14 ± 0.47	10.9 ± 0.5	10.3 ± 0.6	9.94 ± 0.5	9.9 ± 0.6	0.001	0.47
Physical activity (%)	56.7	56.17	50.2	46.4	46.0	45.5	<0.001	<0.001
High (%)^a^	17.4 ± 0.7	18.1 ± 0.7	16.2 ± 0.7	13.9 ± 0.6	14.3 ± 0.8	18.2 ± 0.7	<0.001	0.12
Moderate (%)^a^	14.2 ± 0.8	14.0 ± 0.7	10.8 ± 0.8	9.2 ± 0.6	6.5 ± 0.50	6.4 ± 0.5	<0.001	<0.001
Walking (%)^a^	46.9 ± 0.1	45.5 ± 0.8	40.2 ± 0.9	38.0 ± 0.9	39.9 ± 0.1	37.4 ± 1.0	<0.001	<0.001
Obesity, BMI ≥ 25 (%)	31.6 ± 0.8	31.9 ± 0.8	31.6 ± 0.9	32.5 ± 1.0	32.0 ± 1.0	33.2 ± 0.8	0.57	0.15
Energy intake (kcal)^a^	1902.3 ± 16.6	1928.0 ± 12.8	2068.1 ± 17.1	2053.5 ± 18.1	2003.3 ± 18.3	2057.9 ± 15.0	<0.001	<0.001
Carbohydrate (g/dL)^a^	308.2 ± 2.6	311.6 ± 2.3	332.8 ± 3.0	325.0 ± 2.7	318.4 ± 3.1	320.0 ± 2.6	<0.001	<0.001
Fat (g/dL)^a^	37.5 ± 0.6	39.3 ± 0.5	43.6 ± 0.6	44.0 ± 0.8	44.9 ± 0.8	47.2 ± 0.9	<0.001	<0.001
Fiber (g/dL)^a^	7.5 ± 0.1	7.6 ± 0.1	7.8 ± 0.1	7.4 ± 0.1	7.5 ± 0.1	7.8 ± 0.1	0.006	0.42
Protein (g/dL)^a^	68.2 ± 0.8	69.8 ± 0.7	76.4 ± 0.8	75.5 ± 1.0	74.4 ± 0.9	75.6 ± 0.9	<0.001	<0.001
Vitamin C (mg/dL)^a^	105.8 ± 2.2	106.2 ± 1.9	112.3 ± 2.2	108.8 ± 2.1	112.2 ± 2.8	99.8 ± 2.9	<0.001	0.55
Calcium (mg/dL)^a^	493.9 ± 6.3	500.0 ± 6.2	537.0 ± 6.9	520.5 ± 6.8	509.0 ± 7.7	513.3 ± 6.8	<0.001	0.04
Sodium (mg/dL)^a^	4970.1 ± 62.1	5060.4 ± 55.6	5249.0 ± 67.8	5179.7 ± 75.1	4860.7 ± 71.5	4314.6 ± 59.1	<0.001	<0.001
Serum vitamin D (ng/mL)^a^	19.4 ± 0.3	17.8 ± 0.2	18.0 ± 0.3	17.3 ± 0.2	16.8 ± 0.2	17.2 ± 0.3	<0.001	<0.001

^a^Data are percentage (±SE) and mean (±SE) with appropriate sampling weights. Direct age adjustment of the data was done for the Korean population aged ≥20 years in the year 2005

Table 2 reveals the prevalence of MS and the five components of MS. The overall and sex-specific prevalence (30.8% in men and 26.3% in women in 2013) of MS in the Korean adult population remained stable during the period observed.

**Table 2:** Age-standardized prevalence of the metabolic syndrome and its components in Korea from 2008 to 2013

Year	2008	2009	2010	2011	2012	2013	*P* trend	% changing annually
*N* = 34,587	6,215	6,872	5,661	5,667	5,263	4,909		
Metabolic syndrome								
Unadjusted	26.4 ± 0.9	27.4 ± 0.8	26.4 ± 0.9	28.3 ± 0.9	27.1 ± 1.0	29.0 ± 0.9	0.06	1.5
Age-adjusted	27.5 ± 0.8	28.3 ± 0.7	27.1 ± 0.7	28.6 ± 0.8	27.0 ± 0.9	28.9 ± 0.8	0.50	0.5
Men	27.9 ± 0.1	29.4 ± 0.1	28.0 ± 0.1	29.7 ± 0.1	25.5 ± 0.1	30.8 ± 0.1	0.59	0.6
Women	26.4 ± 0.9	26.5 ± 0.9	25.6 ± 0.8	26.7 ± 0.9	27.6 ± 1.0	26.3 ± 0.9	0.63	0.4
Waist circumference	34.5 ± 1.0	32.5 ± 0.9	31.0 ± 0.9	34.2 ± 1.1	31.1 ± 1.0	30.1 ± 0.9	0.00	−2.3
Men(>90 cm)	27.4 ± 1.2	24.7 ± 1.1	24.4 ± 1.3	27.7 ± 1.3	22.6 ± 1.3	24.8 ± 1.2	0.09	−2.0
Women(>80 cm)	41.0 ± 1.4	39.5 ± 1.2	38.7 ± 1.1	39.9 ± 1.3	38.8 ± 1.4	34.4 ± 1.2	0.002	−2.5
Triglycerides (>150 mg/dL)	28.6 ± 0.8	28.7 ± 0.8	28.5 ± 0.8	28.2 ± 0.8	29.2 ± 0.9	29.4 ± 0.9	0.44	0.5
Men	36.2 ± 1.2	36.8 ± 1.2	37.8 ± 1.3	36.1 ± 1.3	35.7 ± 1.4	37.8 ± 1.4	0.72	0.3
Women	20.5 ± 0.8	20.0 ± 0.8	18.9 ± 0.9	19.9 ± 0.9	22.0 ± 1.0	20.5 ± 0.9	0.36	0.9
HDL cholesterol	43.3 ± 0.9	45.6 ± 0.9	43.0 ± 0.8	42.3 ± 0.1	36.9 ± 0.9	45.8 ± 0.9	0.06	−1.0
Men(<40 mg/dL)	35.3 ± 1.2	36.5 ± 1.2	35.7 ± 1.3	33.9 ± 1.4	26.7 ± 1.3	38.7 ± 1.3	0.15	−1.3
Women(<50 mg/dL)	50.9 ± 1.1	54.2 ± 1.0	50.0 ± 1.0	50.0 ± 1.2	47.0 ± 1.2	52.6 ± 1.1	0.15	−0.8
Blood pressure (≥130/85 mmHg)	31.8 ± 0.7	34.4 ± 0.8	33.5 ± 0.8	36.3 ± 0.8	35.7 ± 1.1	33.4 ± 0.9	0.03	1.3
Men	36.3 ± 1.2	40.7 ± 1.2	38.7 ± 1.3	43.3 ± 1.2	41.5 ± 1.6	39.7 ± 1.3	0.02	1.9
Women	26.9 ± 0.8	27.6 ± 0.8	27.7 ± 0.7	28.8 ± 0.8	29.1 ± 1.1	26.7 ± 0.9	0.56	0.4
Fasting glucose (≥100 mg/dL)	26.8 ± 0.8	27.5 ± 0.8	25.5 ± 0.8	25.1 ± 0.9	27.4 ± 0.9	30.8 ± 0.9	0.00	2.0
Men	30.7 ± 1.2	32.5 ± 1.1	30.4 ± 1.1	30.5 ± 1.2	32.1 ± 1.3	37.0 ± 1.3	0.00	2.7
Women	22.8 ± 0.8	22.5 ± 0.8	20.6 ± 0.9	19.03 ± 1.0	22.6 ± 1.0	24.6 ± 1.0	0.25	1.0

Data are percentage (±SE) with appropriate sampling weights. Direct age adjustment of the data was done for the Korean population aged ≥20 years in the year 2005

*HDL* high density lipoprotein

The most prevalent component of MS was low HDL-C (45.8% in 2013), followed by high BP (33.4%), high FPG (30.8%), abdominal obesity (30.1%), and high TG (29.4%). The prevalence of high TG, high BP, and high FPG was higher in men than in women, whereas the prevalence of low HDL-C and abdominal obesity was higher in women than in men. The prevalence of abdominal obesity decreased in both men and women (annual percent change: −2.0 and −2.5%, respectively); moreover, the decrease was significant only in women. The prevalence of high BP and high FPG (+1.9 and +2.7%, respectively) increased significantly only in men. No significant changes were noted in TG and low HDL-C prevalence between 2008 and 2013.

Table 3 shows the relationship between risk factors and MS using a logistic regression model stratified by sex. In men, the risk of MS increased with age, BMI ≥25 kg/m^2^, current alcohol drinking, current smoking, and vitamin D deficiency. Education and household income were not significantly related to MS risk. In women, the risk of MS increased with age and BMI ≥25 kg/m^2^) and decreased with higher education and higher income. Alcohol drinking, smoking, and vitamin D deficiency were not significantly related to MS risk.

**Table 3:** The association of risk factors and metabolic syndrome

Variables	Men			Women		
	crude OR(95% CI)	adj. OR(95% CI)	P(Wald’s test)	crude OR(95% CI)	adj. OR(95% CI)	*P*(Wald’s test)
Age (years)			<0.001			<0.001
20–29	1	1		1	1	
30–39	2.8 (2.2–3.7)	2.6 (1.9–3.4)	<0.001	2.1 (1.56–2.72)	1.9 (1.5–2.5)	<0.001
40–49	5.4 (4.25–6.85)	5.3 (4.1–6.7)	<0.001	5.0 (3.8–6.5)	3.9 (3.0–5.0)	<0.001
50–59	7.2 (5.7–9.0)	8.5 (6.7–10.9)	<0.001	11.1 (8.6–14.4)	7.2 (5.5–9.3)	<0.001
60–69	8.7 (6.7–10.8)	13.4 (10.3–17.4)	<0.001	25.7 (19.7–33.6)	14.2 (10.8–18.8)	<0.001
70 and over	6.8 (5.3–8.70)	12.0 (9.2–15.8)	<0.001	35.0 (26.9–45.6)	20.5 (15.4–27.4)	<0.001
Obesity (BMI ≥ 25)	5.4 (4.9–6.0)	6.7 (5.9–7.5)	<0.001	7.6 (6.9–8.3)	7.3 (6.6–8.2)	<0.001
Current alcohol drinking	1.1 (0.9–1.2)	1.2 (1.1–1.4)	<0.001	–	–	–
Current smoking	1.0 (0.9–1.1)	1.2 (1.1–1.3)	0.001	0.8 (0.7–0.9)	–	–
Vitamin D deficiency	0.9 (0.8–1.0)	1.1 (1.0–1.3)	0.059	0.52 (0.5–0.6)	–	–
Education						<0.001
Elementary school	1	–	–	1	1	
Middle school	1.1 (0.9–1.2)	–	–	0.43 (0.4–-0.5)	0.7 (0.6–0.9)	<0.001
High school	0.6 (0.5–0.7)	–	–	0.17 (0.16–0.19)	0.67 (0.57–0.79)	<0.001
College and over	0.6 (0.5–0.7)	–	–	0.07 (0.06–0.08)	0.5 (0.4–0.6)	<0.001
Income (Quartile)	–	–	–			<0.001
* Q1*_*lowest*_	1	–	–	1	1	
* Q2*_*medium-lowest*_	0.8 (0.7–0.9)	–	–	0.42 (0.4–0.5)	0.90 (0.8–1.1)	0.221
* Q3*_*Medium highest*_	0.9 (0.7–1.0)	–	–	0.29 (0.26–0.33)	0.8 (0.7–0.9)	0.024
* Q4*_*highest*_	0.8 (0.7–1.0)	–	–	0.22 (0.2–0.3)	0.8 (0.6–0.9)	<0.001
Adjusted R^2^	16.95%		<0.001	29.12%		<0.001

Vitamin D deficiency was defined by 25-hydroxyvitamin D serum concentration ≤20 ng/mL

BMA Model selections by sex: age, obesity (≥25 kg/m^2^), income (quartile), education, smoking (yes/no), drinking (yes/no), physical activity (yes/no), total calorie intake (kcal), carbohydrates (g/dL), fiber (g/dL), sodium (mg/dL), protein (g/dL), fiber (g/dL), vitamin C (mg/dL), fat intake (g/dL), and vitamin D deficiency (yes/no)

## Discussion

A few studies investigated the prevalence of MS in the Korean population, but with little consistency in the MS criteria used. The reported prevalence range of MS varied widely from 1.6 to 29.6% in men and 10.1 to 32.8% in women [8]. The NCEP-ATP III-derived definition of MS was used in this study, and the MS prevalence was similar with that reported in other studies using the same definition [24, 25].

The prevalence of MS increased annually by ~0.6% over 10 years, from 24.9% in 1998 to 31.3% in 2007 based on KNHANES [26]. The increase in the prevalence of the components during 1998–2007 has been explained by a rapid recovery of the economic crisis in 1998, which affected their lifestyle, especially the adoption of westernized diets. Since then, the Korean government and the Korean National Assembly approved laws on health promotion and disease prevention, and one of the main targets of Health Plan 2020 is to reduce smoking, alcohol drinking, and obesity. This policy includes lifestyle interventions, food safety, and public education about healthy eating behaviors and physical activity.

During the period 2008 and 2013, which were covered in the present study, no significant increasing or decreasing trend of MS prevalence was noted. In men, the prevalence of high BP and high FPG increased, whereas that of other components remained constant. Interestingly, the trend prevalence of abdominal obesity among women declined significantly during this period and that of the other four components of MS remained constant.

One of the explanations of increased levels of BP and FPG in men could be that calorie intake and nutrient intake including carbohydrate and fat, which are closely related to high blood sugar and high BP [27], have increased in Korean men and women, although sodium intake significantly decreased. Moreover, physical activity, a preventive factor of high FPG [28] and high BP [29], has decreased.

A decreasing trend of abdominal obesity in women could be expected in consideration of changes in obesity as expressed by BMI. Many studies indicate that obesity is closely associated with hypertension, type 2 diabetes, and hypercholesterolemia [30, 31]. Currently, our understanding of the association between MS and obesity is well established owing to rapidly growing research. One study focusing on multiple products discharged from adipocytes explained the underlying relationship between obesity and MS. The presence of certain products, such as non-esterified fatty acids, inflammatory cytokines, PAI-1, adiponectin, leptin, and resistin, cause an increased risk of developing the components of MS [32].

In the present study, obesity (BMI ≥25.0 kg/m^2^) was significantly related to MS. According to previous investigations, obesity prevalence increased till the year 2007 [33], after which the prevalence of obesity in adults stopped increasing [19, 34], and has even decreased in women; the same trend has also been observed with regard to the prevalence of abdominal obesity [34]. In the population as a whole, obesity prevalence appeared stable during 2008–2013, which may have contributed to the stabilization of MS prevalence.

The prevalence of other factors significantly independently related to MS, such as smoking and alcohol consumption in men and socioeconomic status and education level in women, could also contribute to the stabilization of MS prevalence from 2008–2013.

Associations between smoking and MS and alcohol consumption and MS have been found in many studies. Smoking raises LDL-cholesterol and TGs but reduces HDL-C, causing an increased risk of CVD [35]. Several studies show that smoking may be closely related to high TG, low HDL-C, and abdominal obesity [36, 37]. In addition, in a meta-analysis of 13 prospective studies, long-term observation of the development of MS has revealed smoking to be a contributing factor [38]. Concerning alcohol consumption, a previous study showed that several alcohol-drinking patterns, including: “usual drinking quantity”, “drinking frequency”, “frequency of high-risk drinking”, “frequency of feeling guilty after drinking”, “frequency of inability to stop drinking”, and “frequency of inability to remember after drinking” positively correlated with the prevalence of MS in men [39]. Frequent binge drinking and higher drinking quantity together are indicators of a higher prevalence of MS, and the association strength is thought to be gender-specific [40]. A meta-analysis confirmed that alcohol consumption might be associated with an increased risk of MS [41]. Some biological mechanisms may explain a positive correlation between alcohol drinking and MS risk. Abdominal obesity tends to be more common in excessive drinkers than average drinkers, and this may be a contributing factor in the development of MS [42]. Alcohol drinking also appears to stimulate appetite, thus in part inhibiting the increase in FPG levels [43]. In fact, the prevalence of high FPG in this study was very prevalent among men. During 2008–2013, smoking prevalence significantly decreased, and alcohol consumption remained stable.

In this study, MS risk decreased in women with higher education level and higher income. According to a study by Park et al. [44], women with a higher socioeconomic status are more likely to take care of their health, choose healthy foods, exercise, and undergo regular health check-ups. In contrast, lower socioeconomic status has been linked to health risk behaviors such as smoking, drinking, and lack of physical activity. During the period observed there were no significant changes in socioeconomic status of the Korean population, but changes in education level were significant, with an increase in those with a higher education level (college and over) and a decrease of those with elementary and middle school education.

In this study, vitamin D deficiency was not independently associated with MS. The association between MS and vitamin D deficiency among men was at the edge of statistical significance (*p* = 0.059). The potential involvement of vitamin D in conditions including high BP, cancer, and CVD development and progression have been described in other investigations [45–47]; one study also described an association between vitamin D deficiency and MS [48]. Several studies show similar results. Shokoufeh et al. reported no significant decrease in serum 25-hydroxyvitamin D concentration irrespective of the MS status in an Iranian population [49].

A major strength of our study is that the KNHANES has large, representative samples and observes annual trends. The possible major limitation is that changes in the laboratory methods for measuring serum concentrations of glucose and cholesterol occurred in 2008, which could affect current results of glucose and cholesterol tests; however, a parallel test for quality control of the laboratory analysis conducted by KNHANES confirmed the stability of the blood analysis.

## Conclusions

In conclusion, we found that MS prevalence in Korea is high, but did not follow a significant trend during 2008–2013. Several factors contributed to the stable MS prevalence: on the one hand, increased prevalence of high FPG, high BP, calorie intake, and physical inactivity, and on the other hand, decreased prevalence of abdominal obesity and smoking. Greater awareness of MS and its health consequences can help optimize the treatment of risk factors. Furthermore, risk factors such as smoking, alcohol drinking, obesity, diet, and physical inactivity need to be considered in public health interventions. A multidimensional approach is vital to prevent future increases in MS.

## Abbreviations

BMA: Bayesian model average
BMI: Body mass index
BP: Blood pressure
CI: Confidence interval
CVD: Cardiovascular diseases
FPG: Fasting plasma glucose
HDL-C: High-density lipoprotein cholesterol
KNHANES: Korean National Health and Nutrition Examination Survey
MS: Metabolic syndrome
OR: Odds ratio
TG: Triglyceride
